# A pillar[5]arene-based covalent organic framework with pre-encoded selective host–guest recognition†

**DOI:** 10.1039/d1sc03680g

**Published:** 2021-09-15

**Authors:** Lu Liu, Yiming Hu, Shaofeng Huang, Yinghua Jin, Jingnan Cui, Weitao Gong, Wei Zhang

**Affiliations:** State Key Laboratory of Fine Chemicals, School of Chemical Engineering, Dalian University of Technology Dalian 116024 P. R. China wtgong@dlut.edu.cn; Engineering Laboratory of Boric and Magnesic Functional Material Preparative and Applied Technology Dalian Liaoning Province 116024 P. R. China; Department of Chemistry, University of Colorado Boulder Boulder Colorado 80309 USA wei.zhang@colorado.edu

## Abstract

It is highly desirable to maintain both permanent accessible pores and selective molecular recognition capability of macrocyclic cavitands in the solid state. Integration of well-defined discrete macrocyclic hosts into ordered porous polymeric frameworks (*e.g.*, covalent organic frameworks, COFs) represents a promising strategy to transform many supramolecular chemistry concepts and principles well established in the solution phase into the solid state, which can enable a broad range of practical applications, such as high-efficiency molecular separation, heterogeneous catalysis, and pollution remediation. However, it is still a challenging task to construct macrocycle-embedded COFs. In this work, a novel pillar[5]arene-derived (**P5**) hetero-porous COF, denoted as **P5-COF**, was rationally designed and synthesized. Featuring the unique backbone structure, **P5-COF** exhibited selective adsorption of C_2_H_2_ over C_2_H_4_ and C_2_H_6_, as well as significantly enhanced host–guest binding interaction with paraquat, in comparison with the pillar[5]arene-free COF analog, **Model-COF**. The present work established a new strategy for developing COFs with customizable molecular recognition/separation properties through the bottom-up “pre-porous macrocycle to porous framework” design.

## Abstract 2

A novel pillar[5]arene-derived (**P5**) COF was rationally designed and synthesized, which exhibited superior performance in selective gas adsorption and paraquat binding.

## Graphical abstract

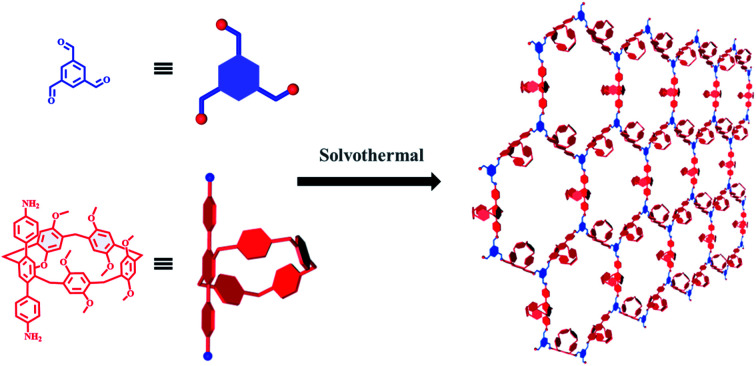

## Introduction

Covalent organic frameworks (COFs) represent a class of crystalline porous materials constructed with diverse organic building blocks *via* covalent bonds.^1–5^ Featuring high surface area, large pore volume, high chemical/thermal stability, and tunable pore topologies, COFs have become a promising platform for gas adsorption and separation,^6–10^ comparable to conventional porous materials, such as zeolites and metal–organic frameworks (MOFs).^11–13^ However, specific supramolecular host–guest interactions, which have been commonly employed in the solution phase to realize selective molecular binding, have rarely been explored in the COF system.^14,15^ One possible way to address this issue is to incorporate macrocyclic cavitands into COFs. Such a “pre-porous macrocycle to porous framework” design would combine the merits of both macrocyclic hosts, with inherent porosity and selective molecular recognition properties, and COFs with stable backbones and a large pore distribution for mass transfer.^16–20^

Pillar[*n*]arenes,^21–23^ a class of macrocyclic cavitands with intrinsic confined pores and selective guest binding capability, are attractive candidates for integration into COFs as host molecules.^24–26^ Although interesting molecular recognition properties of pillar[*n*]arenes in the solution phase have been demonstrated, they generally exhibit poor guest binding capability in the solid state due to their random packing.^27–32^ The opening channels are largely blocked, and the intrinsic cavities are buried inside the solid matrix and not accessible to the guest species.^33,34^ Meanwhile, the supramolecular structures assembled *via* noncovalent interactions are vulnerable and tend to collapse upon guest removal.^35,36^ Therefore, it is challenging to maintain the permanent porosity and selective guest binding properties of pillar[*n*]arenes in the solid state. It is envisioned that the integration of pillar[*n*]arene macrocycles into COFs can overcome the above-mentioned drawbacks. To date, although amorphous porous organic polymers with pillar[*n*]arenes incorporated have been conceived and triggered some interesting applications,^37–44^ to the best of our knowledge, the pillar[*n*]arene-based crystalline COFs have not been realized, likely due to the synthetic challenges.

Herein, we report the rational design and synthesis of a new COF, denoted as **P5-COF**, derived from pre-porous pillar[5]arene-based diamine (**APP5**) and 1,3,5-triformylbenzene (**TFB**) *via* one-pot imine condensation. To demonstrate the critical role of pillar[5]arene macrocycles for the observed selective molecular recognition in the resulting **P5-COF**, a model COF without pillar[5]arene moieties, named **Model-COF** was also prepared. The experiments clearly showed that **P5-COF** exhibits superior performance in selective C_2_H_2_ gas adsorption over C_2_H_4_ and C_2_H_6_, as well as significantly enhanced host–guest binding interaction with paraquat in comparison with **Model-COF**, likely owing to the presence of pillar[5]arene moieties.

## Results and discussion

**Scheme 1:**
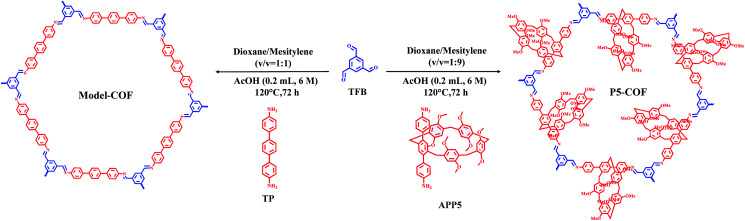
Synthetic routes to **P5-COF** and **Model-COF**.

We synthesized **P5-COF** and **Model-COF** through imine condensation between 1,3,5-triformylbenzene (**TFB**) and **APP5** or 4,4′-diamino-*p*-terphenyl (**TP**) in mixed solvents of dioxane, mesitylene and acetic acid aqueous solution at 120 °C for 3 days, respectively (Scheme 1). **P5-COF** and **Model-COF** were characterized by FT-IR spectroscopy, ^13^C CP-MAS NMR spectroscopy, TGA, SEM and PXRD analysis. The FT-IR spectra of both **P5-COF** and **Model-COF** show the disappearance of the aldehyde peak at 1693 cm^−1^ and amine peaks at 3449 and 3356 cm^−1^. Meanwhile, the appearance of the –C=N stretching peak at 1624 cm^−1^ supports the formation of imine bonds *via* the condensation of aldehyde and primary amine (Fig. S3 and S4†).

**Fig. 1:**
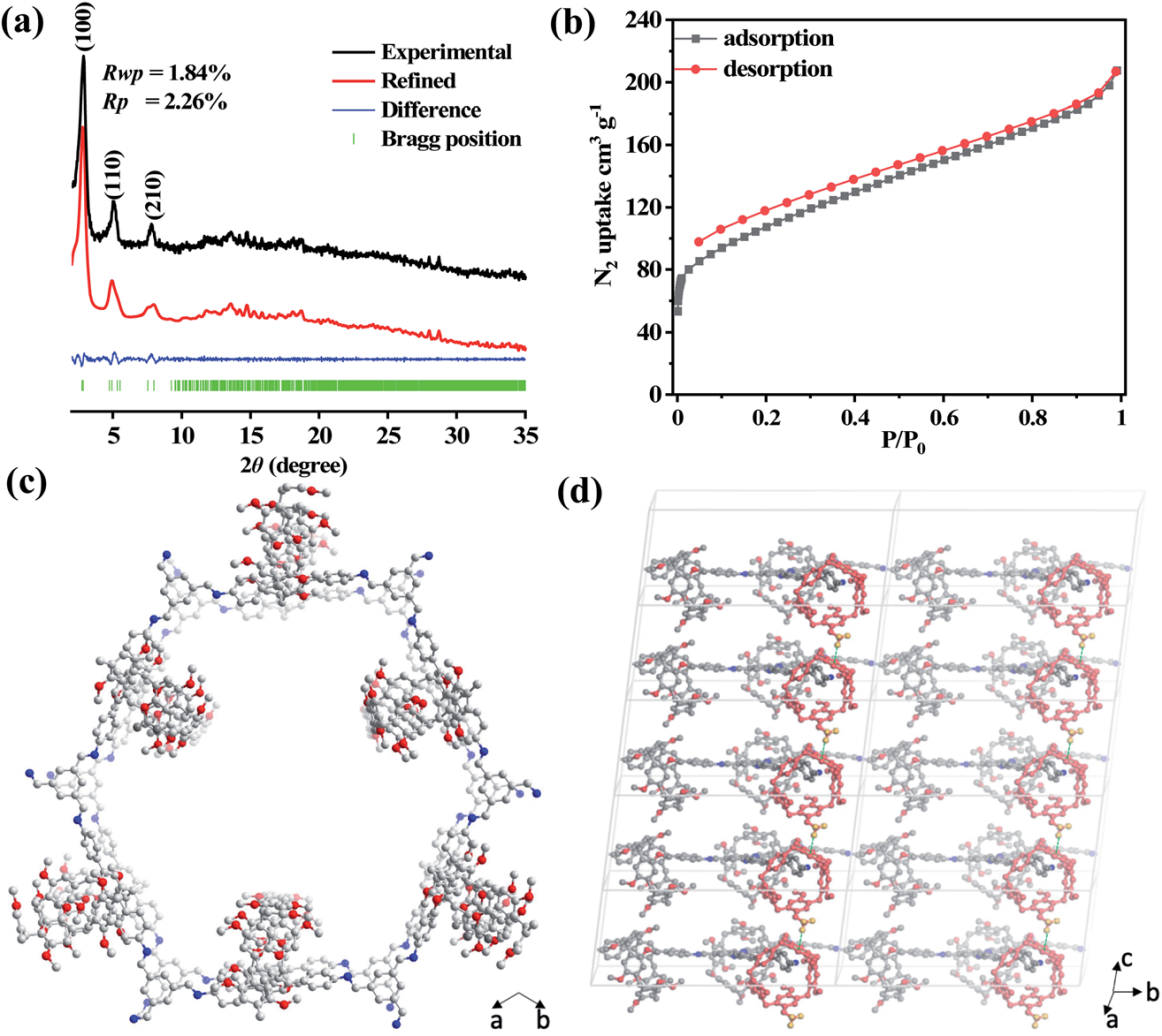
(a) Experimental and simulated PXRD patterns of **P5-COF**; (b) N_2_ adsorption isotherms of **P5-COF** at 77 K; (c and d) top view (c) and side view (d) of **P5-COF** in the AA stacking model. The green dash indicates the CH–π stabilizing interactions.

The cross-polarization magic-angle spinning (CP-MAS) NMR spectrum of **P5-COF** shows a peak around 157 ppm, corresponding to the carbon atom of the –C=N bond, and signals at 122–138 ppm, which can be assigned to the carbon atoms of the phenyl groups (Fig. S5 and S6†). The peaks with the chemical shifts around 55 and 37 ppm were assigned to the methoxy carbon and benzyl carbon in pillar[5]arenes, respectively (Fig. S5†). Thus, the solid-state NMR data support the structure of **P5-COF**. The thermogravimetric analysis (TGA) of the materials showed good thermal stability with a decomposition temperature of 420 °C for **Model-COF** and 400 °C for **P5-COF** (Fig. S7†). Furthermore, the morphologies of the two COFs were characterized by SEM and TEM (Fig. S8 and S9†). **P5-COF** shows irregular particles with rather smooth surfaces, whereas **Model-COF** shows a moss-like surface morphology. The good crystallinity of **P5-COF** was confirmed by powder X-ray diffraction (PXRD) (Fig. 1a). To determine the packing structure of **P5-COF**, the eclipsed and staggered stacking conformations of the pillar[5]arene structure were simulated. In order to construct the most energy stable packing pattern, we examined the single crystal packing mode of pillar[5]arene small molecules. We found that CH–π interactions between pillar[5]arene molecules largely stabilize the crystal structure.^45^ Thus, we introduced similar CH–π interactions when incorporating the pillar[5]arene moieties along the *z* axis to minimize the system energy. Accordingly, we built a **P5-COF** structure in the *P*3 space group with Pawley-refined unit cell parameters *a* = 37.2308 Å, *b* = 35.9837 Å, *c* = 13.3034 Å, *α* = 86.93°, *β* = 89.76°, and *γ* = 116.91° (Fig. 1c and d). The PXRD patterns of **P5-COF** exhibit three intense peaks at 2.88°, 5.04° and 7.77°, which correspond to the (100), (110) and (210) reflections, respectively. The Pawley-refined PXRD patterns agree well with the experimental results with *R*_p_ and *R*_wp_ values of 1.84% and 2.26%, respectively (Fig. 1a). The as-obtained **Model-COF** also exhibits good crystallinity, showing a strong and sharp peak at 2.42° in the PXRD spectrum, which could be assigned to the (100) Bragg degree reflection. The peaks at 4.69° and 5.36° were assigned to the (110) and (200) reflections, respectively (Fig. S10†). To explore the stability of these frameworks, **P5-COF** and **Model-COF** were immersed in different organic solvents and aqueous solutions (EtOH, THF, DMF, water, aqueous HCl (2 M), and NaOH (2 M)) for 24 hours. The PXRD data obtained before and after treatment barely showed any difference, which indicates high chemical stability of both COFs (Fig. S11†).

Nitrogen sorption isotherms at 77 K were then measured to evaluate the porosity of the COFs. Both the isotherms of **P5-COF** and **Model-COF** present type-IV adsorption. The BET surface areas of **P5-COF** and **Model-COF** are calculated to be 381 m^2^ g^−1^ and 134 m^2^ g^−1^, respectively (Fig. 1b and S12†). The pore size distributions (PSDs) were evaluated with the quenched solid density functional theory (QSDFT) equilibrium model. **Model-COF** exhibits a pore size distribution in the range of 2.0–3.2 nm, which is in good agreement with the literature report (Fig. S14†).^46^**P5-COF** exhibits two major pores with the sizes of 0.78 nm and 1.43 nm, which likely result from the intrinsic pillarene macrocycles and internal aligned channels in the COF. It is worth noting that the pore diameter of 0.78 nm is in agreement with the cavity size of pillar[5]arenes, which supports the presence of the permanent pillar[5] cavity in the COF.^47^ The calculated total pore volumes of **Model-COF** and **P5-COF** are 0.53 and 0.32 cm^3^ g^−1^, respectively (Table S1†).

The unique pillar-shaped architectures and rigid π-rich cavities of pillar[*n*]arenes make them high affinity hosts for paraquat and other electron-deficient molecules. However, conventionally such guest-binding behavior has been mostly observed in the homogeneous solution.^48–52^ In the solid state, due to random molecular aggregation, many intrinsic pores are blocked, thus dramatically decreasing the guest-binding capacity.^53^ By integrating pillar[5]arenes into ordered COFs, we envisioned that such long-standing problems in their guest binding in a heterogeneous environment can be overcome.

**Fig. 2:**
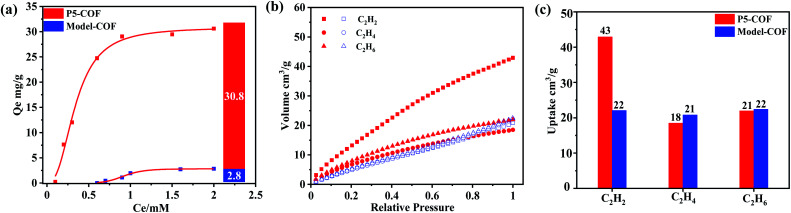
(a) Langmuir adsorption isotherms of paraquat by **P5-COF** and **Model-COF**. Inset: the comparison of the paraquat adsorption capacity of **P5-COF** and **Model-COF**; (b) gas adsorption isotherms of **P5-COF** (red) and **Model-COF** (blue) at 273 K; (c) comparison of gas adsorption uptakes of **P5-COF** and **Model-COF** at 273 K.

To explore the binding behavior of the **P5-COF** solid material toward paraquat in a heterogeneous environment, the absorption isotherm experiments with various concentrations of paraquat (0–0.2 mM) at room temperature were conducted. As shown in Fig. 2a, the Langmuir equilibrium isotherm model of paraquat matches very well with a good distribution coefficient. We found that **P5-COF** exhibits a good adsorption capacity for paraquat (*q*_max_ = 31 mg g^−1^). Furthermore, as shown in Fig. S16,† the adsorption efficiency remained >80% even after five cycles of the adsorption study, and **P5-COF** retained its chemical structure and crystallinity after five cycles based on PXRD and FT-IR spectra. By contrast, **Model-COF** did not show any obvious adsorption of paraquat, which is consistent with our assumption that the adsorbed paraquat by **P5-COF** was due to the host–guest interaction between the pillar[5]arene units of **P5-COF** and paraquat. These results support that when a well-defined molecular porous structure is introduced into an ordered porous polymer, the host–guest binding behavior (*e.g.*, binding selectivity, affinity, *etc.*) could be transferred into the polymer matrix; hence, **P5-COF** bridged the gap between the organic porous polymers and the supramolecular macrocycles as pre-porous building blocks.

Given the encouraging results of the paraquat adsorption study, we next explored the gas adsorption properties of **P5-COF**. Hydrocarbons play a significant role in the petrochemical industry and have been considered as important energy sources due to the high value of these hydrocarbons as chemical feedstock and their wide applications. In this study, C_2_H_2_, C_2_H_4_, and C_2_H_6_ were chosen as the probe molecules to examine the gas adsorption properties. All measurements were conducted at 1 bar and 273 K. The uptakes of C_2_H_6_, C_2_H_4_, and C_2_H_2_ by **Model-COF** were 22, 21, and 22 cm^3^ g^−1^, respectively. At the same temperature and pressure, the uptakes of C_2_H_6_, C_2_H_4_, and C_2_H_2_ by **P5-COF** were 21, 18, and 43 cm^3^ g^−1^, respectively. It should be noted that the uptake of C_2_H_2_ by **P5-COF**was increased by 110%, while the uptake of C_2_H_6_ and C_2_H_4_ was decreased slightly when compared to the amount adsorbed by **Model-COF** (Fig. 2c). This difference in acetylene uptake is presumably due to the more favorable interaction between pillar[5]arene moieties and acetylene molecules through hydrogen bonding and π–π, or C–H–O interactions,^54–57^ thus showing the potential of **P5-COF** in selective separation of acetylene. We also calculated the natural selectivity of C_2_H_2_ over C_2_H_4_, which is an important parameter in the industrial process of ethylene purification. **P5-COF** showed a natural selectivity of 2.4, which is comparable to that of many other organic frameworks.^58–60^ The selectivity calculated by the ideal adsorption solution theory (IAST) method is 3.2 with a 50 : 50 feeding mixture at a total pressure of 1 bar and 273 K. However, as shown in Table S2,† the IAST selectivity of C_2_H_2_/C_2_H_4_ is comparable to that of the well-known MOFs such as series ZJNU-14 (2.05), HUST-5 (1.8), and HUST-6 (1.42). In sharp contrast, the **Model-COF** showed nearly no selectivity for C_2_H_2_ over C_2_H_4_ at 273 K. Considering that these two COFs have a similar C_2_H_4_ uptake amount, the higher C_2_H_2_ uptake of **P5-COF** mainly results from the interaction between the adsorbent and adsorbate.

## Conclusions

In summary, we have synthesized, for the first time, a pillar[5]arene-based COF (**P5-COF**), which showed heteroporosity and notable C_2_H_2_ and paraquat adsorption capabilities. More importantly, **P5-COF** exhibits superior performance in selective C_2_H_2_ gas adsorption over C_2_H_4_ and C_2_H_6_, as well as significantly enhanced adsorption of paraquat compared with that of pillar[5]arene-free **Model-COF**. We believe that the macrocyclic structural features of pillar[5]arenes, the connectivity of their intrinsic cavities, and pore size of the framework play a critical role in the performance characteristics of **P5-COF**. Our study opens new possibilities for developing novel COF materials with specific guest binding capability by integrating host moieties into porous frameworks and harnessing their supramolecular host–guest binding interactions in the solid state, which conventionally has been possible only in the solution phase.

## Data availability

All the data have been included in the ESI.†

## Author contributions

Conceptualization and supervision: W. Gong and W. Zhang; synthesis and characterization (XRD, PXRD, TGA, IR, adsorption): L. Liu, Y. Hu, and S. Huang; writing, reviewing, & editing: L. Liu, S. Huang, Y. Hu, Y. Jin, W. Gong and W. Zhang. The ideal adsorption solution theory (IAST) method calculations: S. Huang, all authors proof-read, provided comments, and approved the final version of this manuscript.

## Conflicts of interest

There are no conflicts to declare.

## Supplementary Material

SC-012-D1SC03680G-s001
