# Supplementary material for: A pillar[5]arene-based covalent organic framework with pre-encoded selective host–guest recognition
Source: Chem Sci. 2021 Sep 15;12(40):13316–20. doi: 10.1039/d1sc03680g (PMC8528016; doi:10.1039/d1sc03680g)
Supplement: SC-012-D1SC03680G-s001 [file SC-012-D1SC03680G-s001.pdf]

Electronic Supplementary Material (ESI) for Chemical Science.
This journal is © The Royal Society of Chemistry 2021

Electronic Supplementary Information for

Pillar[5]arene-Based Covalent Organic Framework with Pre-encoded Selective Host-guest Recognition

Lu Liu,[a,c] Yiming Hu,[c] Shaofeng Huang,[c] Yinghua Jin,[c] Jingnan Cui,[a] Weitao Gong, * [a,b] Wei Zhang * [c]

[a.] *State Key Laboratory of Fine Chemicals, School of Chemical Engineering, Dalian University of Technology, Dalian 116024, P. R. China.*

[b.] *Engineering Laboratory of Boric and Magnesic Functional Material Preparative and Applied Technology, Dalian, Liaoning Province, 116024, P. R. China.*

[c.] *Department of Chemistry, University of Colorado Boulder, Boulder, Colorado 80309, United States.*

1. Materials and general methods **2**
2. Experimental procedures **3**
Scheme S1. Synthetic scheme of APP5 .... **3**
Synthesis of compound APP5: **3**
Fig. S1 ^{1}H NMR spectrum of compound **APP5** 4
Fig. S2 ^{13}C NMR spectrum of compound **APP5** 4
Synthesis of COFs **5**
Synthesis of P5-COF. 5
Synthesis of Model-COF 5
Fig. S3 FT-IR spectra of **TFB**, **APP5**, and **P5-COF**. 6
Fig. S4 FT-IR spectra of **TFB**, **TP** , and **Model-COF**. 6
Fig. S5 ^{13}C CP/MAS NMR spectrum of **P5-COF**. 7
Fig. S6 ^{13}C CP/MAS NMR spectrum of **Model-COF**. 7
Fig. S7 Thermogravimetric analysis of **Model-COF** and **P5-COF** 8
Fig. S8 SEM images of **Model-COF** and **P5-COF**. 8
Fig. S9 TEM images of **P5-COF** and **Model-COF**. 9
Fig. S10 PXRD analysis of **Model-COF**. 9
Fig. S11 PXRD pattern of **P5-COF** and **Model-COF** after immersed in different solution 10
Fig. S12 N_2 Adsorption Isotherms of **Model-COF**. 10
Fig. S13 Top view and side view of **Model-COF** 11
Fig. S14 The pore size distribution of **P5-COF** and **Model-COF**. 11
Table S1. The pore properties of P5-COF and Model-COF. **11**
Standard Curve of UV adsorption vs. paraquat concentration: **12**
Fig. S16 Stablily test of **P5-COF** for Paraquat 12
Fig. S17 Gas adsorption isotherms of **P5-COF** and **Model-COF** 13
Table S2. Comparison of gas adsorption properties of P5-COF with other reported MOFs. **13**
References: ***13***

1. Materials and general methods

All commercial chemicals are of analytical grade and were used without further purification. Compound **P5-OTf** and **APP5** were synthesized following previously reported procedures [1,2].

NMR spectra were taken on Bruker 300 and Inova 500 spectrometers. $CHCl_3$ (7.26 ppm) was used as an internal reference in ^1H NMR, and $CDCl_3$ (77.16 ppm) for ^{13}C NMR spectra. NMR data is reported in the following order: chemical shift, multiplicity (s, singlet; d, doublet; t, triplet; q, quartet; m, multiplet), coupling constants (*J*, Hz), number of protons. Solid-state cross-polarization magic-angle spinning (CP/MAS) NMR spectra were recorded on an Inova 400 NMR spectrometer. Powder X-Ray Diffraction (PXRD) was obtained from Inel CPS 120 diffraction system, using monochromated Cu Kα (λ=1.542 Å) radiation.

The FT-IR spectra of starting materials and COFs were obtained from Agilent Technologies Cary 630 FT-IR. Thermogravimetric analyses (TGA) were performed on Mettler Toledo TGA/DSC3+ instrument by heating the samples under nitrogen atmosphere at a heating rate of 5 °C min^{-1} within the temperature range of 30-800 °C.

The Quantachrome Autosorb ASiQ automated gas sorption analyzer was used to measure N_2 adsorption isotherm. The samples were heated at 120 °C and kept at this temperature for 24 h under vacuum for activation. Ultra high purity grade (99.999% purity) N_2, C_2H_2, C_2H_4, C_2H_6, and gas regulators were used for all free space corrections and measurements. For the gas adsorption measurement, the temperatures were controlled by using a refrigerated bath of liquid N_2 (77 K), ice water (273K) and water (295 K). The pore size distribution was calculated with quenched solid density functional theory (QSDFT) and cylindrical pore model based on the nitrogen sorption isotherms at 77 K using Quantachrome AsiQwin program. The morphology of the samples were characterized by a Tescan Vega3 scanning electron microscope (SEM).

Adsorption capacity:

The polymer was added into H_2O of paraquat (3 mL) with an initial concentration of 0.06 mM, 0.07 mM, 0.08 mM, 0.09 mM, 0.1 mM, 0.16 mM, and 0.2 mM. The suspension was stirred for 12 h and then filtered. The concentration of paraquat in the

filtrate was determined with UV-Vis spectroscopy (257 nm).

The adsorption data were fitted with Langmuir isotherm model using the following equation:

$$q_e = \frac{q_{max} b c_e}{1 + b c_e}$$

Where q_e (mg g^{-1}) is the amount of pollutant adsorbed at equilibrium, C_e (mM) is the residual pollutant concentration at equilibrium, q_{max} (mg g^{-1}) is the maximum adsorption capacity of pollutants, and b is the equilibrium constant.

2. Experimental procedures

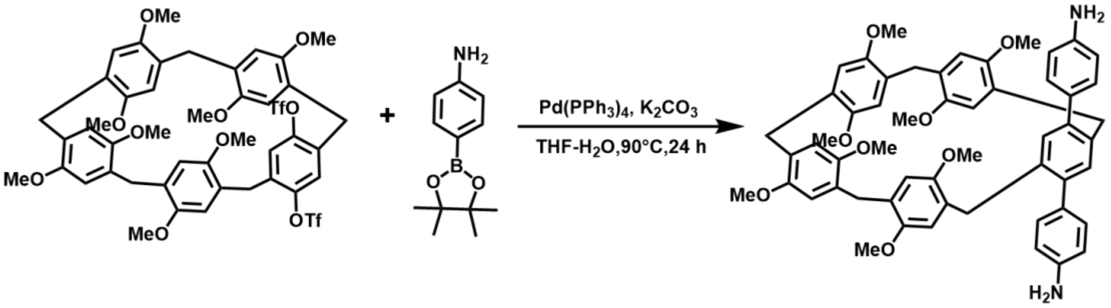

Scheme S1. Synthetic scheme of **APP5**.

Synthesis of compound APP5:

P5-OTf (1.70 g, 2.0 mmol), 4-aminophenylboronic acid pinacol ester (0.99 g, 4.5 mmol), potassium carbonate (2.76 g, 20 mmol), $Pd(PPh_3)_4$ (0.15 g, 0.1 mmol) and H_2O (10 mL) were dispersed in THF (40 mL). The mixture was degassed with nitrogen and stirred at 90 °C overnight. The mixture was concentrated under reduced pressure, then purified by column chromatography to get **APP5** as a white powder (1.04 g, 60.0%): ^1H NMR (400 MHz, $CDCl_3$, 298K) δ 6.93 (s, 2H), 6.79 (d, *J* = 8.1 Hz, 4H), 6.72 (d, *J* = 9.9 Hz, 4H), 6.55 (s, 2H), 6.48 (d, *J* = 8.4 Hz, 4H), 5.94 (s, 2H), 3.91-3.87 (m, 4H), 3.81-3.74 (m, 6H), 3.68 (s, 6H), 3.61-3.56 (m, 4H), 3.53 (s, 6H), 3.39 (s, 6H), 3.35 (s, 6H); ^{13}C NMR (75 MHz, $CDCl_3$) δ 150.95, 150.81, 150.67, 150.48, 144.91, 139.80, 136.46, 132.68, 132.34, 114.64, 114.25, 114.14, 114.09, 113.62, 56.03, 55.99, 55.73, 55.48, 33.07, 29.73, 29.25; HRMS: m/z calcd. for $[M+H]^+$ $C_{55}H_{57}N_2O_8$:873.4115; found 873.4114.

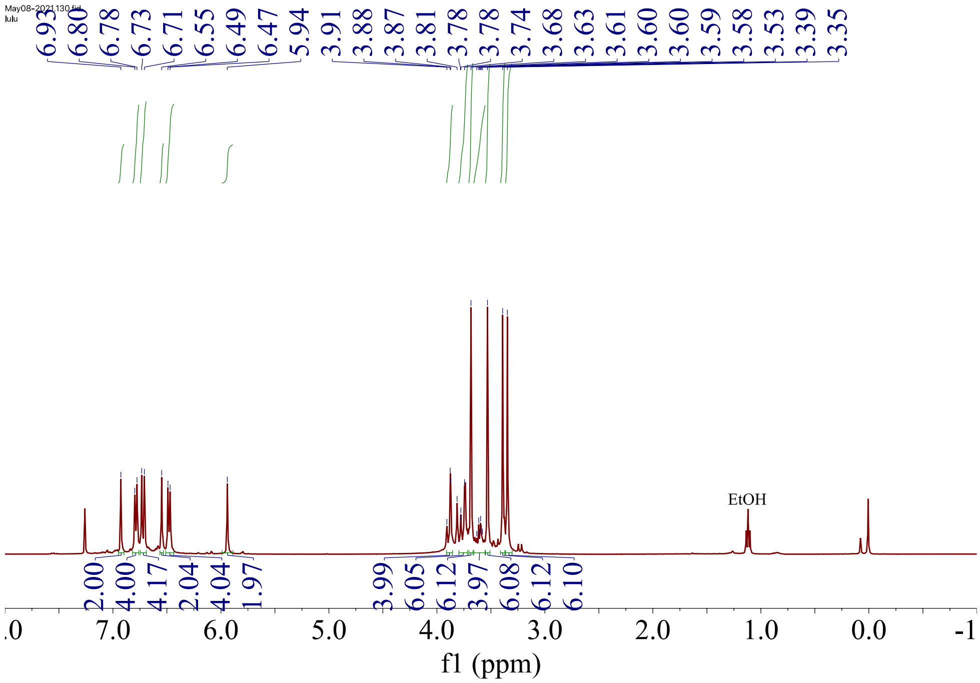

Fig. S1 ^{1}H NMR spectrum of compound **APP5** in $CDCl_3$ on 400 MHz.

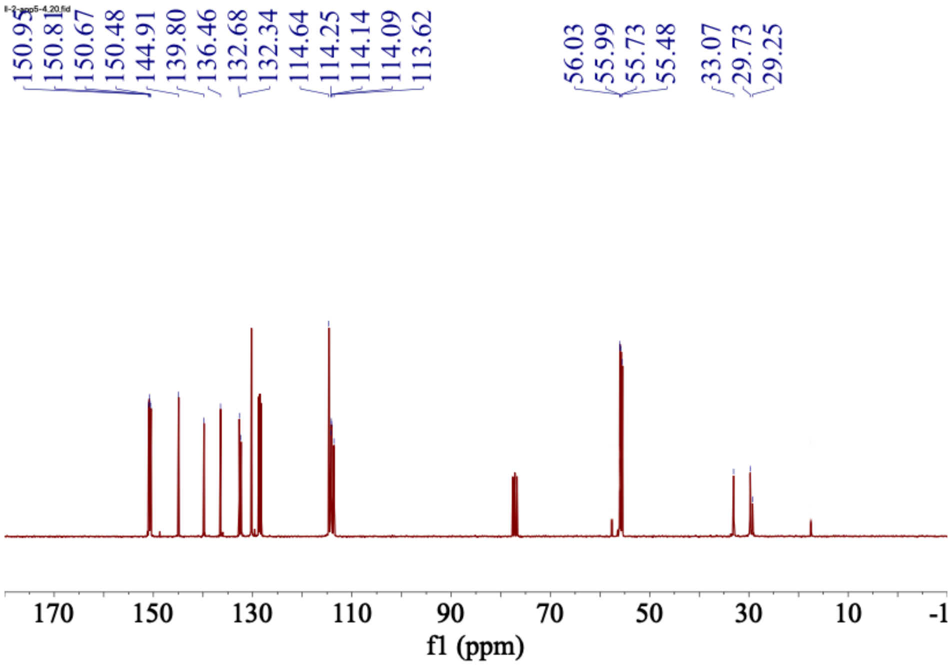

Fig. S2 ^{13}C NMR spectrum of compound **APP5** in $CDCl_3$ on 75 MHz.

Synthesis of COFs.

Synthesis of P5-COF.

An ampule was charged with 1,3,5-triformylbenzene (**TFB**) (8.0 mg, 0.050 mmol) and **APP5** (65 mg, 0.075 mmol) in a mixture of dioxane (0.10 mL), mesitylene (0.90 mL), and an aqueous solution of AcOH (0.20 mL, 6 M). The ampule was frozen at 77 K in liquid nitrogen and evacuated to the internal pressure of ~100 mTorr. Then the ampule was sealed with flame and heated at 120 ℃ for 3 days without any stirring and disturbance. The precipitate was collected by vacuum filtration, washed with THF under Soxhlet extraction for 48 h and further dried in vacuum at 120 °C. **P5-COF** was obtained as a yellow powder (57 mg, 78%): Anal. Calcd for **P5-COF** $(C_{384}H_{360}N_{12}O_{48})_n$: C, 78.03; H, 6.14; N, 2.84; Found: C, 76.38; H, 6.08; N, 3.00.

Synthesis of Model-COF

An ampule was charged with 1,3,5-triformylbenzene (**TFB**) (8.0 mg, 0.050 mmol) and 4,4'-Diamino-*p*-terphenyl (**TP**) (20 mg, 0.075 mmol) in a mixture of dioxane (0.50 mL), mesitylene (0.50 mL), and an aqueous solution of AcOH (0.20 mL, 6 M). The ampule was frozen at 77 K in liquid nitrogen and evacuated to the internal pressure of ~100 mTorr. Then the ampule was sealed with flame and heated at 120 ℃ for 3 days without any stirring and disturbance. The precipitate was collected by vacuum filtration, washed with THF under Soxhlet extraction for 48 h, and further dried under vacuum at 120 °C. **Model-COF** was obtained as a yellow powder (18 mg, 63%): Anal. Calcd for **Model-COF** $(C_{163}H_{123}N_{12})_n$: C, 87.02; H, 5.51; N, 7.47 Found: C, 82.01; H, 5.12; N, 7.66.

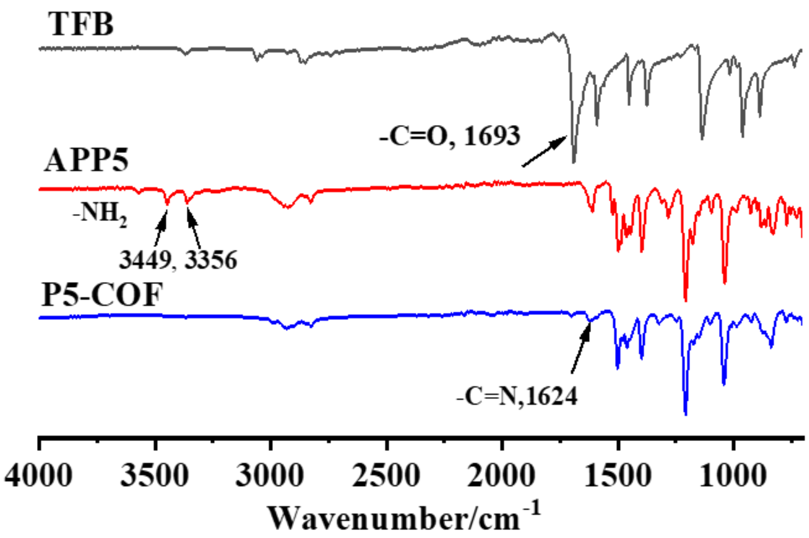

Fig. S3 FT-IR spectra of **TFB** (black), **APP5** (red), and **P5-COF** (blue).

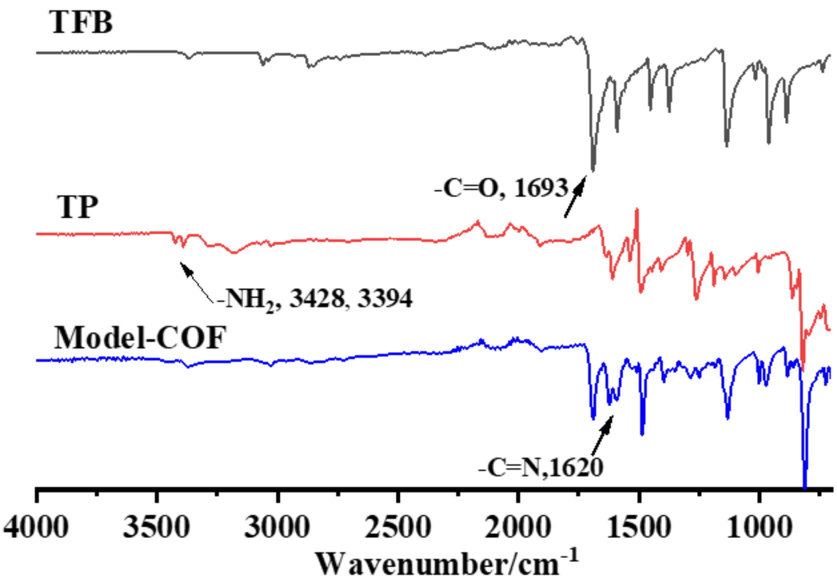

Fig. S4 FT-IR spectra of **TFB** (black), **TP** (red), and **Model-COF** (blue).

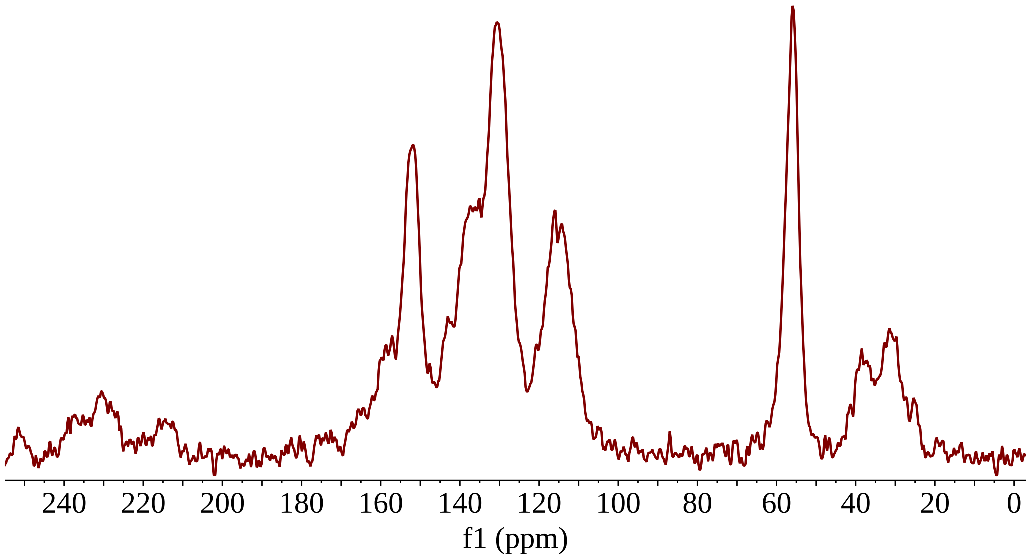

Fig. S5 ^{13}C CP/MAS NMR spectrum of **P5-COF**.

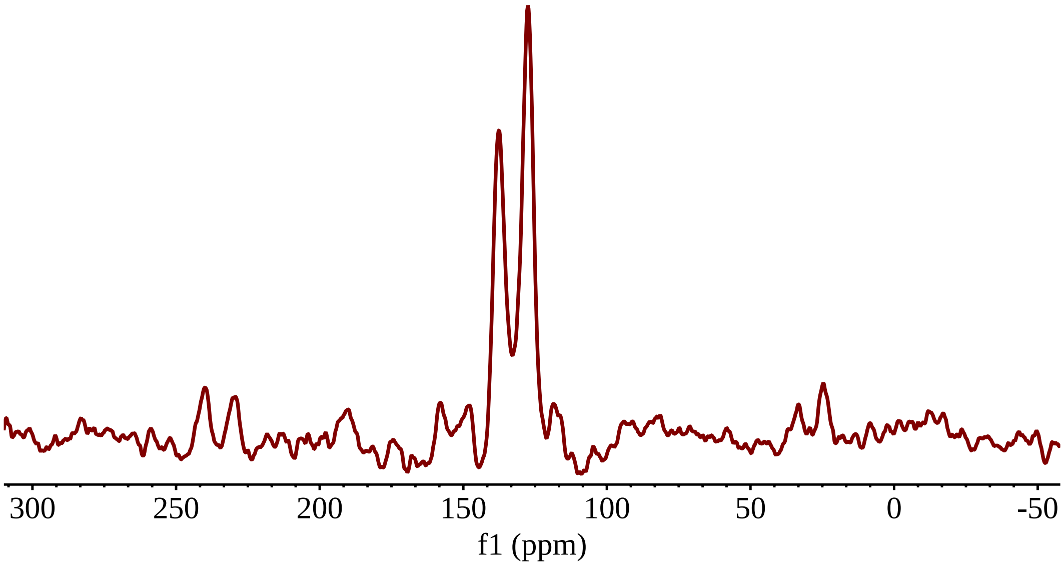

Fig. S6 ^{13}C CP/MAS NMR spectrum of **Model-COF**.

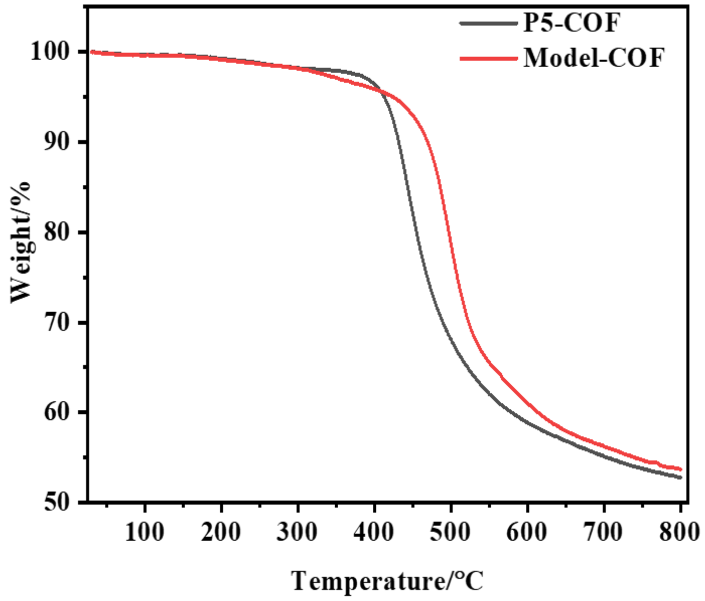

Fig. S7 Thermogravimetric analysis of **Model-COF** (red) and **P5-COF** (black) networks under N_2 atmosphere.

(a) (b)

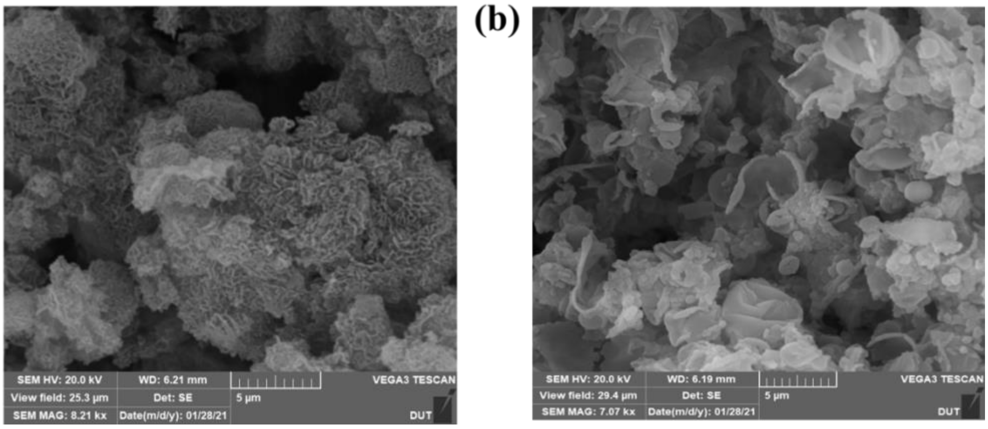

Fig. S8 SEM images of **Model-COF** (a) and **P5-COF** (b).

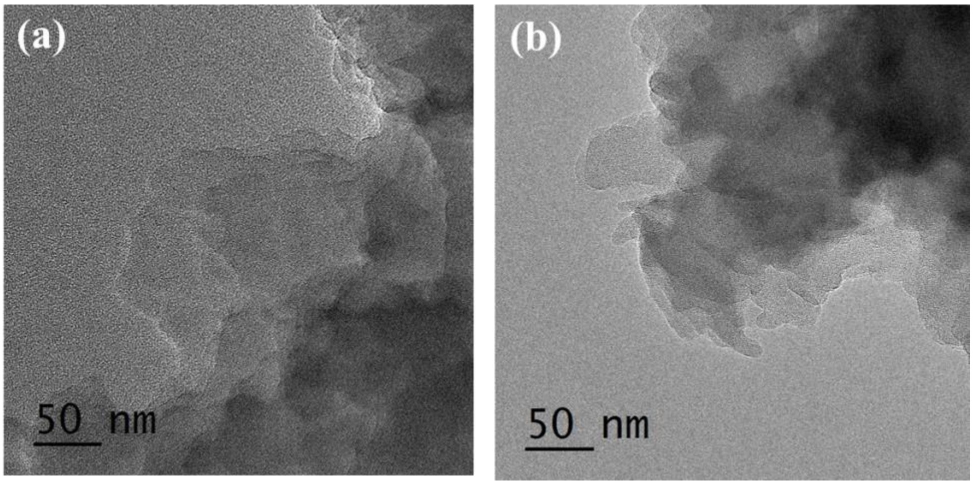

Fig. S9 TEM images of **P5-COF** (a) and **Model-COF** (b).

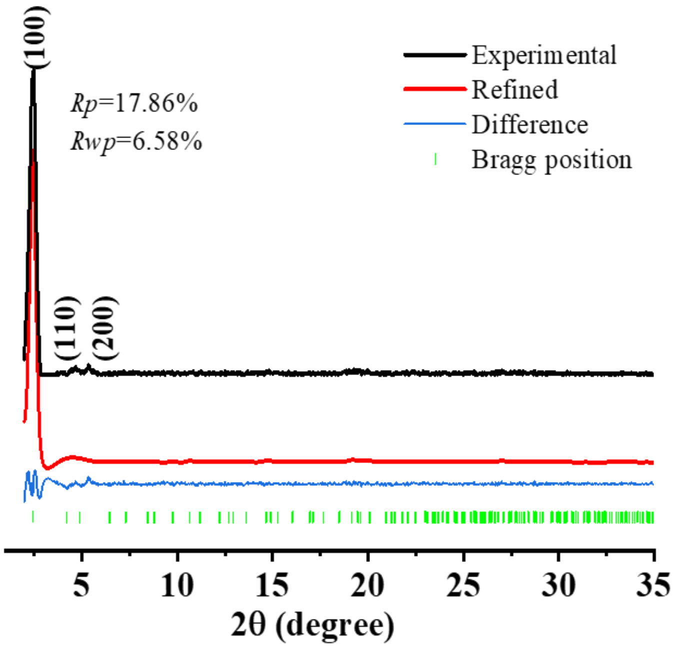

Fig. S10 PXRD analysis of **Model-COF**.

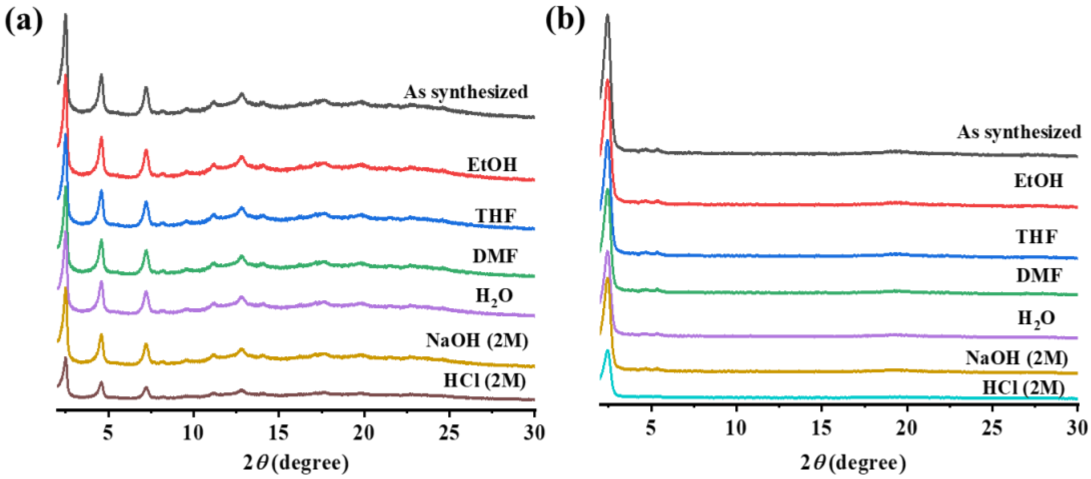

Fig. S11 PXRD pattern of **P5-COF** (a) and **Model-COF** (b) after immersed in different solution for 24h.

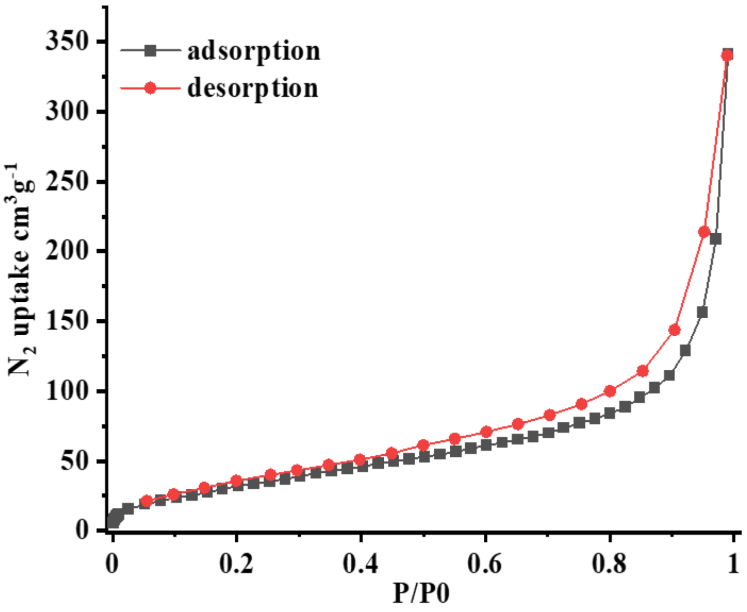

Fig. S12 N_2 Adsorption Isotherms of **Model-COF**.

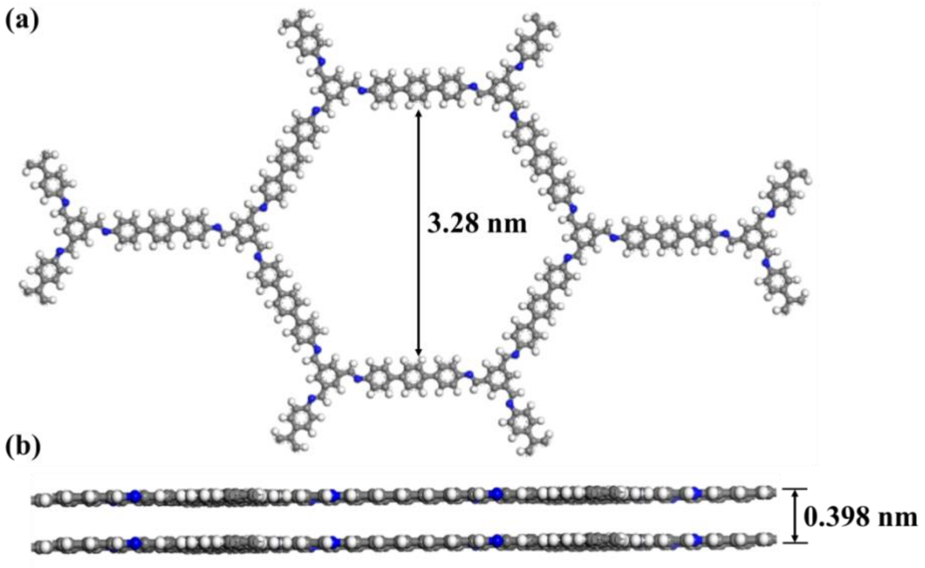

Fig. S13 Top view (a) and side view (b) of **Model-COF** in AA stacking model.

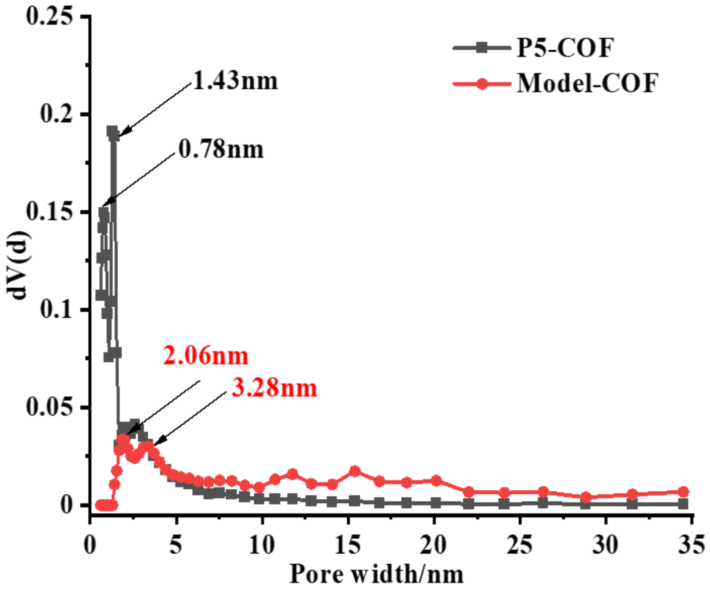

Fig. S14 The pore size distribution of **P5-COF** and **Model-COF**.

Table S1. The porosity of **P5-COF** and **Model-COF**.

Sample	S_{BET} ($m^2\ g^{-1}$)	S_{Micro} ($m^2\ g^{-1}$)	V_{Micro} ($cm^3\ g^{-1}$)	V_{Total} ($cm^3\ g^{-1}$)	Pore size (nm)
**Model-COF**	134.0	0	0	0.53	2.06/3.28
**P5-COF**	380.0	139.2	0.063	0.32	0.78/1.43

Calibration curve of UV adsorption vs. paraquat concentration:

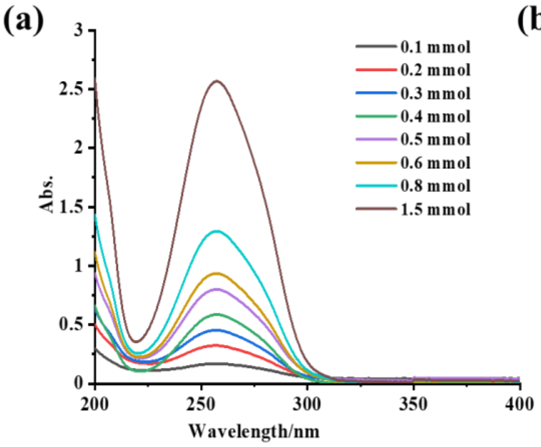

Fig. S15 UV-Vis spectra of paraquat at different concentrations (a); Calibration curve plotted based on the absorbance at 257 nm (b).

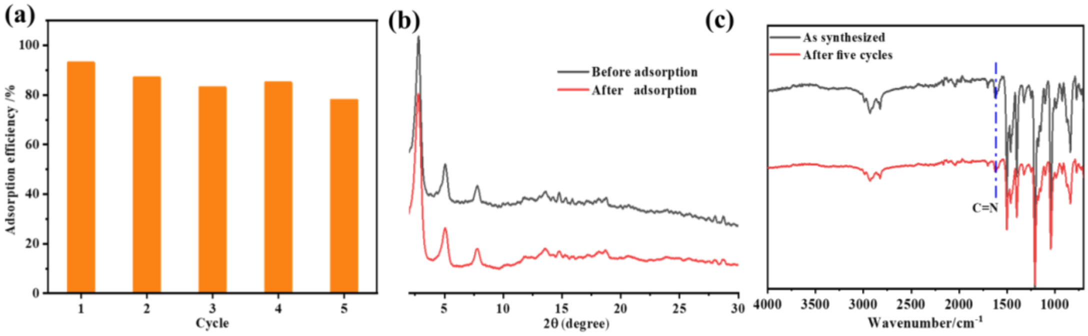

Fig. S16 (a) Recyclability of **P5-COF** for paraquat adsorption, (b) PXRD pattern of **P5-COF** before and after five cycles of adsorption study, (c) FT-IR spectra of **P5-COF** before and after five cycles of adsorption study.

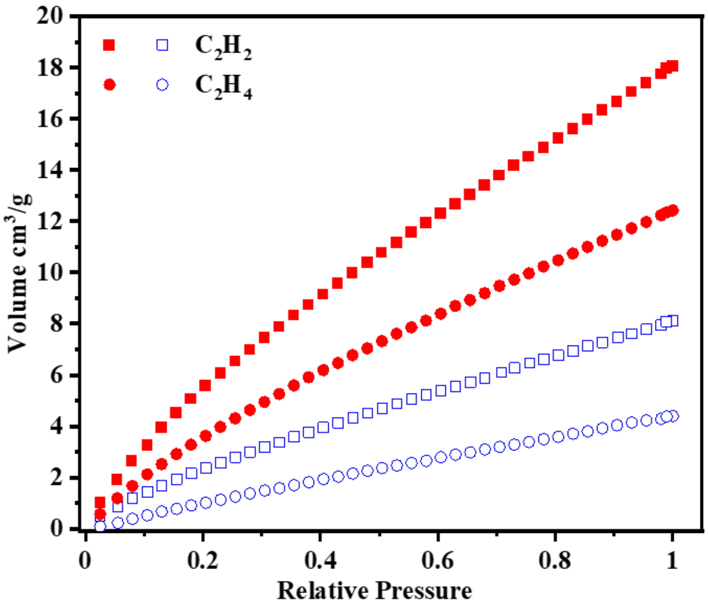

Fig. S17 Gas adsorption isotherms of **P5-COF** (red) and **Model-COF** (blue) at 295 K.

Table S2. Comparison of gas adsorption properties of **P5-COF** with other reported MOFs.

Adsorbents	C_2H_2 uptake (cc g^{-1})	C_2H_4 uptake (cc g^{-1})	C_2H_2/C_2H_4 Selectivity (50:50)	Condition	Ref.
NPU-1	114	94	1.4	298K	J. Am. Chem. Soc., 2021, 143, 1485 - 1492.
NPU-2	90	77.2	1.25	298K	
NPU-3	57.7	49.7	1.32	298K	
SIFSIX-3-Ni	73.9	39.2	5.98	298K	Science, 2016, 353, 141 - 144.
SIFSIX-2-Cu	120.5	45.2	4.95	298K	
SIFSIX-1-Cu	190.4	92	8.37	298K	
$Ni_3(pzdc)_2(7Hade)_2$	52.9	33.1	130	298K	Angew. Chem. Int. Ed. 2020, 59, 18927 - 18932.
HUST-5	50	38	1.8	273K	J. Mater Chem. A, 2020, 8, 2083 - 2089.
HUST-6	78	57	1.42		
P5-COF	43	18	3.2	273K	This work
ZJNU-14	109.8	86.9	2.05	273K	Dalton Trans., 2020, 49, 15672- 15681.
P5-SOF	36	23	20	273K	Chem. Commun., 2017, 53, 6409 - 6412.

References:

[1] S. Zhang, X.Li, W. Gong, T. Sun, Z. Wang and G. Ning, Pillar[5]arene-Derived Microporous Polyaminal Networks with Enhanced Uptake Performance for CO_2 and Iodine, *Ind. Eng. Chem. Res.,* 2020, **59**, 3269-3278.

[2] L. Xu, Z. Wang, R. Wang, L. Wang, X. He, H. Jiang, H. Tang, D. Cao and B. Z. Tang, A Conjugated Polymeric Supramolecular Network with Aggregation-Induced Emission Enhancement: An Efficient Light-Harvesting System with an Ultrahigh Antenna Effect, *Angew. Chem. Int. Ed.,* 2020, ***59***, 9908-9913.
